# A growth model driven by curvature reproduces geometric features of arboreal termite nests

**DOI:** 10.1098/rsif.2020.0093

**Published:** 2020-07-22

**Authors:** G. Facchini, A. Lazarescu, A. Perna, S. Douady

**Affiliations:** 1 Life Sciences Department, University of Roehampton, London, UK; 2 Institut de Recherche en Mathématique et Physique, UCLouvain, Louvain-la-Neuve, Belgium; 3 Laboratoire Matière et Systèmes Complexes, Université Paris Diderot, Paris, France

**Keywords:** social insects, stigmergy, pattern formation, self-organization, complex systems

## Abstract

We present a simple three-dimensional model to describe the autonomous expansion of a substrate whose growth is driven by the local mean curvature of its surface. The model aims to reproduce the nest construction process in arboreal *Nasutitermes* termites, whose cooperation may similarly be mediated by the shape of the structure they are walking on, for example focusing the building activity of termites where local mean curvature is high. We adopt a phase-field model where the nest is described by one continuous scalar field and its growth is governed by a single nonlinear equation with one adjustable parameter *d*. When *d* is large enough the equation is linearly unstable and fairly reproduces a growth process in which the initial walls expand, branch and merge, while progressively invading all the available space, which is consistent with the intricate structures of real nests. Interestingly, the linear problem associated with our growth equation is analogous to the buckling of a thin elastic plate under symmetric in-plane compression, which is also known to produce rich patterns through nonlinear and secondary instabilities. We validated our model by collecting nests of two species of arboreal *Nasutitermes* from the field and imaging their structure with a micro-computed tomography scanner. We found a strong resemblance between real and simulated nests, characterized by the emergence of a characteristic length scale and by the abundance of saddle-shaped surfaces with zero-mean curvature, which validates the choice of the driving mechanism of our growth model.

## 1. Introduction

Self-organized growth phenomena are ubiquitous in physics, societies and biology, with examples ranging from dunes and ripples in the sand, to crystals and cities, to developing plants and embryos (see [1] for a review). All such phenomena are associated with autonomous processes in which a substrate increases its size while simultaneously developing a characteristic shape.

In some systems, growth is driven directly by volumetric expansion. This process is common, for instance, in living tissues, where cell proliferation can happen both at the surface and inside the volume. In other systems, growth is driven mainly by accretion processes, whereby new material is added or removed only at the surface of the growing object (crystals, concretions, corals and shells are examples of this latter type of growth) [2]. As a consequence, accretive growth is inevitably related to the dynamics of the interfaces between the growing substrate and the external environment. Importantly the shape of the interface can contribute to focusing the growth-driving quantity at specific positions, thus causing the onset of positive feedback loops that produce fingering and tip-splitting. The growth-driving quantity itself can be different in different systems. For example it could be pressure, as in the seminal work of Saffman & Taylor [3]—where one fluid invades a more viscous one—or it could be the concentration of a protein as in the more recent work of Clément *et al.* [4], which was aimed at modelling the branching morphogenesis of lungs. At small length scales, negative feedbacks (viscous dissipation, capillarity) stabilize the system and fix the typical scale of the final pattern [5]. Growth is then often a self-sustained process where the form of the substrate is the main ingredient as well as the main outcome of the growth process.

A particular example of self-organized accretive growth is nest building by social insects. At first sight, collective nest building by social insects may look different from the examples above because the transport and deposition of building material is mediated by active agents—the insects—that are capable of multiple regulations of activity, and of complex movement patterns. In fact, there is extensive evidence that nest building can be influenced by a number of factors, including insects responding to environmental gradients [6], crowding [7], the insects using their own body as a template [8] and individual abilities such as path integration [9] (see [10] for a review). Yet, while insect movement itself can follow complex rules, the decisions that insects take of adding or removing pellets at a particular place obey simple ‘stigmergic’ principles whereby the local configuration of the environment is the main driver that determines the probability of deposition and collection of material [11,12]. When insect density is high, the effect of such stigmergic regulations is likely to dominate the other factors and the nest growth resembles an accretive growth. In this continuum approach, the construction process should be described by a Turing-like system of coupled equations, one for each of the interacting fields—for example, density of agents, pheromones and CO_2_ concentration—which are constrained by the shape of the structure under construction.

However, the stigmergic nature of interactions between insects and the growing substrate justifies an alternative and simplified approach where only the growing substrate is modelled explicitly and the nest grows by itself as a function of its own shape, similarly to a crystal or a tissue.

**Figure 1.**
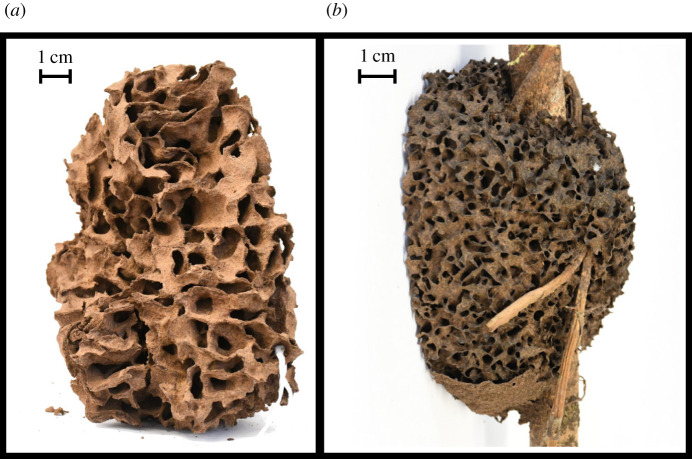
Fragments of nests built by *Nasutitermes walkeri* (*a*) and *Nasutitermes ephratae* (*b*).

Here, we focus in particular on modelling the nests built by arboreal termites of the genus *Nasutitermes*. These are lightweight—but robust—structures that are usually built up on trees, typically around a branch or a tree fork. Their structure is often isotropic and very homogeneous, to the point that different parts of the nest can hardly be distinguished; galleries show a typical length scale and there are no chambers or other specialized structures (figure 1). As a consequence, these nests resemble a continuum substrate with a coherent morphology, which makes them particularly suitable to be treated as the result of accretive growth. Our task is to identify a relevant and minimal ‘stigmergic function’ that summarizes the results of the interactions of termites with the building material (i.e. the local rules of growth and remodelling of the built structure) and that is sufficient to produce structures comparable to those observed in the real nests.

The actual construction process is difficult to observe, as termites concentrate their building activity in short and unpredictable bursts. The only available descriptions of termite behaviour during nest expansions come from the laboratory experiments of Jones [13] (see also [14]). Jones observed that construction always happens by deposition of material on the edge of existing walls, and that these edges progressively bend, split and merge in order to form an intricate matrix, where the local surface of the walls often resembles a saddle. The observation that wall edges concentrate the building activity indicates that the growth of the structure is enhanced where the mean curvature is high; nest growth would instead be inhibited in regions where mean curvature is close to zero, as is suggested by the appearance and relative stability of saddle-shaped surfaces.

The idea that curvature can guide termite aggregation and building behaviour was investigated much more recently in experiments by Calovi *et al.* [15], who demonstrated the existence of a positive correlation between termite activity and surface curvature in *Macrotermes michaelseni* termites (an African termite species not too closely related to *Nasutitermes*).

A role of surface curvature in directing building behaviour is also consistent with the observations by Fouquet *et al.* [16], where elevated structures act as substrate aggregation points, and by Green *et al.* [17], who report that pellet depositions happen mostly at the edges of excavation sites. However, we want to stress here that identifying local curvature as a guiding factor in termite construction does not equate to stating that termites respond to curvature directly. Termites could impregnate the building material with a ‘cement pheromone’ that stimulates pellet depositions, or respond directly to the gradient of humidity evaporating from the built structure. While there is scarce evidence in support for a specific role of ‘cement pheromones’ in termite building [18], and these putative pheromones often covary with the water content of the building material [19], our reasoning remains unchanged as long as these stimuli correlate with the local surface curvature of the structures.

Based on these observations, we develop a model of surface evolution that depends directly on surface curvature. The model is reminiscent of gradient growth processes, where the local curvature of the surface concentrates gradients and fuels instabilities. In these processes, the instability is strong and results in separate branches that compete together [20], and cannot fuse [21]. Here the nest surfaces are tightly interconnected so we have to find a new way of expressing this growth, allowing both separation and fusion.

## 2. Growth model

A large set of growth phenomena can be identified with the dynamics of the interface that divides the growing substrate from the medium that supports the growth. We are then confronted with a two-phase problem where boundary conditions change in time and take the form of a curve or a surface, depending on whether the geometry of the problem is highly confined in one direction or fully three-dimensional like our nests. Whenever this curve or surface largely deviates from a planar front, it is quite challenging to treat the problem analytically, and predictions can be done only locally. Implementing a time-evolving boundary condition in three dimensions is also difficult and would certainly require additional computing resources compared with a regular domain. As an alternative approach, here we adopt a phase-field model [22], or diffuse interface method, which consists in replacing the substrate and the medium with a continuous phase endowed with a scalar field *f* which is defined everywhere in the considered volume and determines the presence of the first or the second entity according to a threshold value. The frontier between the two phases can then be easily retrieved as an iso-contour of *f* in the considered volume. As long as *f* is continuous, the geometry of the frontier can also be obtained from *f* via differentiation [23], the growth model is then extremely simple and consists of only one differential equation for the field *f*. The growth equation should be nonlinear because we want the growth process to saturate at some point, and we also want to impose that new walls are only built adjacent to pre-existing ones, i.e. we want to describe an accretion process. We define our scalar field *f* in the interval [0, 1], and assume that the actual surface of the walls corresponds to the iso-surface *f* = *f*_0_; for example, *f*_0_ = 0.5. We will then say that a point ***x*** is inside the wall whenever *f*(***x***) > *f*_0_ and outside otherwise. If the nest growth is a function of its own shape, the growth equation must take the form

2.1 $$\frac{\partial f}{\partial t}=A(f),$$

where the operator A is meant to mimic the main features of the growth process as they are inferred from nest observations, and we construct it as follows:

2.2 $$A(f)=-f^{\alpha }{\left(1-f\right)}^{\beta }\cdot d(\nabla \cdot \hat{n})-\Delta (\nabla \cdot \hat{n}),$$

where the constant *d* is strictly positive and the equation is presented in a non-dimensional form. The unit vector $\hat{n}$ is the normal to the local iso-surface and is given by $\hat{n}=\nabla f/|\nabla f|$; for example, $\hat{n}(x_{0})=(\nabla f/|\nabla f|){|}_{x_{0}}$ will be the normal to the iso-surface *f*(***x***) = *f*(***x***_0_) at the point ***x***_0_.

The first term of the equation represents the actual growth and can be decomposed into two factors: a differential operator $-d(\nabla \cdot \hat{n})$ and a nonlinear kernel *f*^*α*^(1 − *f*)^*β*^. The first factor is precisely the mean curvature of the local iso-surface multiplied by a constant, and is intended to mimic how local curvature enhances the building or digging activity: the field *f* increases (decreases) whenever the local curvature is positive (negative). The second factor vanishes at *f* = 0, 1 and is maximum at *f* = *f*_0_ = *α*/(*α* + *β*); it is meant to damp any variation of *f* far from the iso-surface *f* = *f*_0_, which is the only accessible space for our walking agents: there cannot be any growth in the empty space far from the pre-existing nest nor deep inside a pre-existing wall. Finally, the exponents *α* and *β* are intended to grasp the possible lack of symmetry between the walls and the empty space, but this scenario is not discussed in the present work, i.e. we will always consider *α* = *β* = 1.

The second term in equation (2.2) is proportional to the diffusion of the mean curvature, which consequently compensates the growth term whenever the local curvature is too high. Technically, this is a dissipative term which provides a cut-off at the highest wavelength and is necessary to produce a meaningful pattern, as is highlighted below in the linear analysis. Phenomenologically, there are several reasons to introduce this term. On the one hand, the growth of a wall happens (field and experimental observations) by the sequential deposition of faecal pellets and soil/sand particles of finite size, thus we can expect the existence of a minimum scale in the spatial structure. On the other hand, one can expect that, although it is mostly organized, the contributions of thousands of agents must show a certain level of randomness whose average effect likely produces some diffusion. Finally, an explicit justification can be found in the experimental observations of Jones [24], which include a smoothing process among the tasks performed by individual workers.

Now that a growth equation is defined we want to make a further approximation. In fact the curvature operator $\nabla \cdot \hat{n}$ is highly non-analytical (and nonlinear) while we would like our equation to be as simple as possible for both analytical and numerical purposes. In the hypothesis that |∇f| is almost constant in the vicinity of *f* = 0.5, one can approximate $\nabla \cdot \hat{n}\propto \Delta f$. In addition we take *α* = *β* = 1, which fixes the maximum of the nonlinear kernel and the contour of the actual nest at *f*_0_ = 0.5. Thus, our simplified growth equation reads

2.3 $$\frac{\partial f}{\partial t}=-f(1-f)d\Delta f-\Delta^{2}f,$$

where Δ is the standard Laplacian or diffusion operator and Δ^2^ is the bi-Laplacian operator that commonly enters elasticity problems [25] and was recently included in a phenomenological model for vegetation patterns [26]. One observes that Δ*f* appears here with the opposite sign of a common diffusion equation, that is, the first term in equation (2.3) is anti-diffusive, while the second term is a diffusive term of higher order or a hyper-diffusion. Note that the difference in the derivative order of the two operators is the key ingredient for pattern formation, as will be shown below through linear analysis. Finally, one may remark that equation (2.3) does not conserve the total mass (or volume) because of the nonlinear pre-factor *f*(1 − *f*). We stress that these two elements are necessary to produce an organized structure like our termite nests, as already observed by Deneubourg [27].

### 2.1. Linear analysis

In its simplified form (2.3), the growth equation can be easily linearized around *f*_0_ = 0.5 and solved in Fourier space with solutions of the form $f=f_{0}+\hat{\;f}\exp\left[\text{i}k\cdot x+\sigma t\right]$, which give the following dispersion relation:

2.4 $$\sigma (k)=\tilde{\;\;f}dk^{2}-k^{4},$$

where for simplicity we wrote |***k***| = *k* and $\;\tilde{\;f}=f_{0}(1-f_{0})=0.25$. The dispersion relation is shown in electronic supplementary material, figure S1. The parameter *d* is strictly positive, which means that *σ*(*k*) changes sign at $k_{0}={\left(\;\tilde{\;f}d\right)}^{1/2}$ and has a local maximum at $k_{\max}={\left(\;\tilde{\;f}d/2\right)}^{1/2}$: equation (2.3) is linearly unstable with the most unstable length at *λ*_max_ = 2*π*/*k*_max_ while all the small-scale disturbances beyond the cut-off length *λ*_0_ = 2*π*/*k*_0_ are damped. Note that *k*_0_ > 0 independently of *d*, thus the system is formally always unstable. Nonetheless, a threshold is fixed by the smallest *k* that can appear in a given domain of size *L*, which is *k* = 2*π*/*L*. The instability threshold *d*_*c*_ is then fixed by the size *L* of the domain, with $d_{c}={\left(2\pi /L\right)}^{2}/\;\tilde{\;f}$ being the marginal condition. One also remarks that the cut-off length *λ*_0_ is only $\sqrt{2}$ smaller than *λ*_max_. This means that, while inspired by the granular nature of the building material, the dissipative term smooths away any details smaller than wall thickness. As a consequence, our model cannot reproduce any internal porosity of the walls, a parameter that termites could control through the choice of the building material [28] and that plays an important role for ventilation [29] of termite mounds.

Interestingly, in two dimensions, the same eigenvalue problem arises when studying the buckling modes of a thin plate under symmetric in-plane compression [30]. The stationary two-dimensional version of equation (2.3) coincides with a particular case of Föppl–von Kármán equations that were discovered more than a century ago [31]. Nonetheless, a very similar problem has gathered new attention in recent decades to explain delamination patterns occurring in multi-layered material where the coating material does not have the same elastic properties as the underlying substrate [32–34]. Notably a similar mechanism was also invoked to explain the formation of Miura-ori folding patterns that approximately appear in nature; for example, in insect wings and blooming leaves [35]. In most cases, emerging patterns significantly differ from the planar solution (i.e. the solution we chose at the beginning of this section) and likely arise from secondary instabilities [36], which rather select a superposition of planar waves that reflects the invariance of their orientation at the instability onset.

## 3. Numerical simulations

We simulate equation (2.3) on a cubic grid with a finite difference code written in Python and automatically parallelized with the Numba compiler. The largest domain we considered is 256 × 256 × 256, where a growth simulation can be accomplished within about 5000 cpu hours. Both Laplacian and bi-Laplacian operators are implemented with a first-order explicit Euler scheme. Two different boundary conditions were considered: in the first configuration boundary conditions are triply periodic, while in the second one lateral faces of the simulation box are still periodic but Dirichlet boundary conditions (i.e. *f* = const.) are imposed on the bottom and top faces. Initial conditions are also varied. In the simplest case, the whole simulation domain is initiated with white noise, mainly to characterize the long-term (or quasi-stationary) solutions of the equation within the bulk. Alternatively, we initiate simulations with part of the volume copied from a previous simulation where a distinct pattern has already appeared, while the remaining part of the domain is set to zero. In this case, we also forbid any evolution for all the points that are initially above a certain threshold which maintains the initial seed unchanged. This configuration is intended to mimic the expansion process of a pre-existing nest, or a new one which starts from an arbitrary shaped support (in the following, we will refer to that as a nest-like seed).

## 4. Results

**Figure 2.**
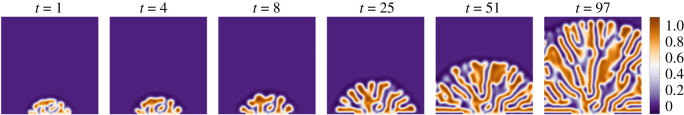
Time sequence of a vertical cut of the field *f* for a simulation starting with a noisy half-sphere at the bottom of the simulation domain. Boundary conditions are periodic at the lateral boundaries, and *f* = const. at the top and bottom boundaries. The time is normalized by the growth rate of the most unstable mode in electronic supplementary material, figure S1.

**Figure 3.**
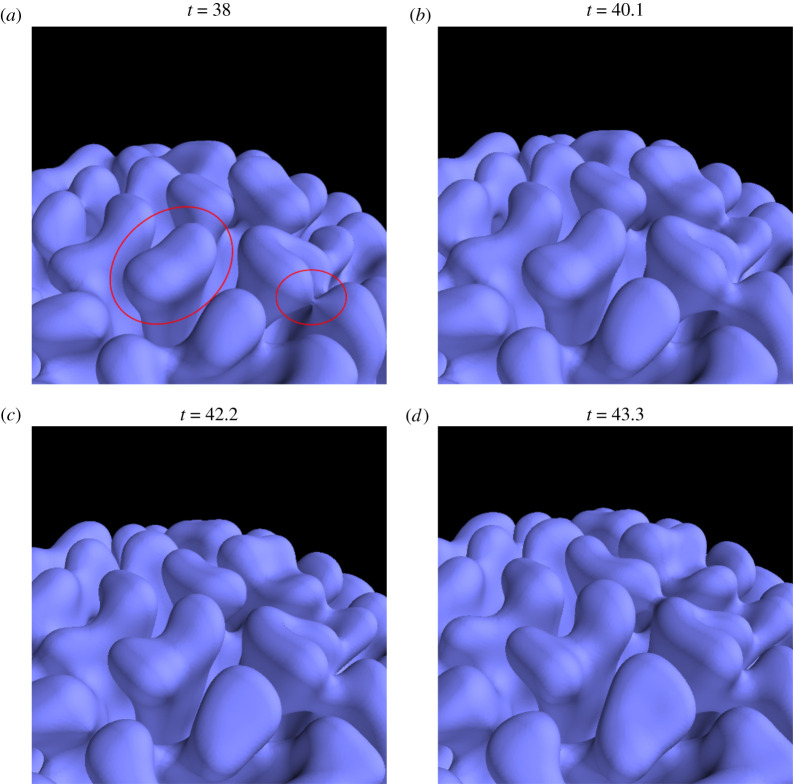
Evolution of the *f* = 0.5 iso-surface over a period of five time scales: one recognizes at least one branching episode and one merging episode (red circles). The simulation is the same as the one described in figure 2.

As a first result, we report that our model reproduces a growth process. In figure 2, we show the evolution of the field *f* for a simulation initiated with a nest-like seed with the shape of a half-sphere at the bottom of an empty domain (*f* = 0), and time is normalized using the growth rate of the most unstable mode $\sigma_{\max}=f_{0}^{2}d^{2}/4$ (see electronic supplementary material, figure S1). One observes that the initial seed grows, and progressively invades the empty space; even more interestingly we notice that far from the interface between the empty space and the growing nest the field *f* is essentially stationary (for example, there is little rearrangement of the pre-existing nest). This indicates that equation (2.3) describes an accretion phenomenon happening at the interface between the growing walls and the empty space, even if we did not implement any drying or ageing effect and the field *f* could, in principle, change everywhere and at any time. Finally, we remark that the growing interface frequently branches and merges, as is shown in figure 3. Note that merging and branching episodes cover a few time units, which confirms that local growth is correctly described by linear analysis and our time scale is well chosen.

**Figure 4.**
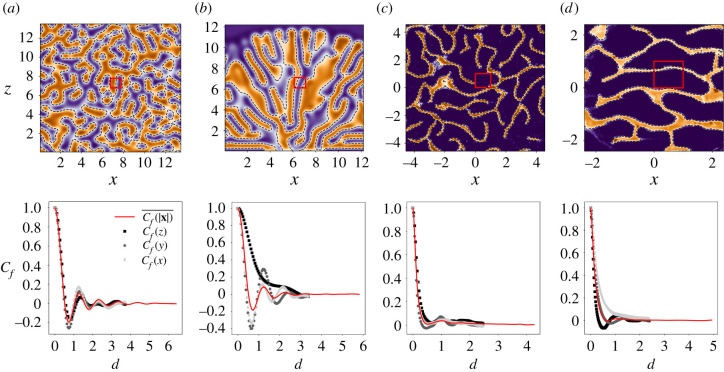
Top: Cross section of the nest volume for two simulations (*a*,*b*) and two computed tomography scans of real samples (*c*,*d*). The walls are in orange and the empty space is in blue. The red square indicates the peak of the three-dimensional FFT over the whole volume. Bottom: Auto-correlation functions *C*_*f*_, each one corresponding to the nest above it. Symbols correspond to the value of *C*_*f*_ when computed only in one Cartesian direction, while the solid lines correspond to the average value of *C*_*f*_ over all possible directions. For each nest, lengths are rescaled by the corresponding FFT peak.

Secondly, we observe that in the growing pattern of figure 2 a characteristic length scale appears which is the typical distance between two walls. We report that a similar result was obtained for all the initial conditions and boundary conditions we considered. In figure 4*a*–*d* (top), we show a two-dimensional slice of the *f* field for two different simulations (*a,b*), where (*a*) has triply periodic boundary conditions and was initiated with white noise everywhere, while (*b*) is the one already presented in figure 2. Both simulations were considered at large time, namely at *t* = 64 time scales for simulation (*a*) and *t* = 93 time scales for simulation (*b*). To track the existence of a characteristic length scale in our objects we perform a three-dimensional fast Fourier transform (FFT), which is shown in electronic supplementary material, figure S2. The dominant frequency of the FFT was then obtained and superimposed (squares) on the patterns in figure 4 as a comparison. We remark that the dominant length indicated by the FFT is consistent with the observed patterns. Finally, we note that the emerging lengths are fairly consistent with the most unstable length indicated by linear analysis, as shown in electronic supplementary material, figure S1. Nevertheless, the observed patterns significantly differ from the planar solutions we consider in §2.1, and appear to be modulated in multiple directions. To quantify this feature, we compute the auto-correlation functions *C*_*f*_(*x*), *C*_*f*_(*y*), *C*_*f*_(*z*) in the three Cartesian directions (symbols) and the function *C*_*f*_(|***x***|) averaged over all directions (solid line), with all the distances rescaled by the typical length given by the FFT. The results are shown at the bottom of figure 4*a*–*d*. First, one observes that in all the diagrams *C*_*f*_(|***x***|) shows a first maximum around *d* = 1, which is consistent with FFT analysis. Then we remark that in simulation (*a*) all the directional correlation functions present the same peak as *C*_*f*_(|***x***|), which indicates that the observed pattern is highly isotropic, while in simulation (*b*) there is no peak in the vertical correlation function *C*_*f*_(*z*), which indicates that there is low modulation in the vertical direction. Indeed, all the simulations we performed ultimately show a similar sheet-like anisotropy. This happens after a very slow process of rearrangement, which usually takes tens or even hundreds of time scales after the pattern has invaded the entire domain. Our interpretation is that this particular pattern is always selected by nonlinear interactions or secondary instabilities similar to those observed in elastic plates [35,36], but different initial conditions can either enhance or not enhance this effect, determining the time at which this pattern takes over.

## 5. Comparison with real nests

We collected nest fragments of two different species of *Nasutitermes*, namely *Nasutitermes walkeri* from the Sydney area (Australia) and *Nasutitermes ephratae* from Guyana; two of these are shown in figure 1. The walls of these nests are sometimes infra-millimetric, thus a correct reconstruction of their structure was possible only with the use of a micro-computed tomography (micro-CT) scanner whose resolution was set to 0.1 mm. A typical scan consists of a stack of images, where higher intensity corresponds to the walls and lower intensity to the empty space, analogously to the field *f* that we consider in our model and simulations. We repeated our scale analysis on two CT scans of fragments belonging to real nests of the species *N. ephratae* (*c*) and *N. walkeri* (*d*), which are reported in figure 4. As for simulated nests, the FFT of real nests indicates a dominant length scale which is consistent with the observed distance between the walls (see electronic supplementary material, figure S2). Looking at the auto-correlation functions one sees that fragment (*d*) is scarcely modulated in the horizontal direction, which is consistent with the observed pattern, while fragment (*c*) appears fully isotropic. Note that for these fragments vertical and horizontal directions were completely arbitrary. Indeed different degrees of isotropy or different local structures can be encountered in different species and even within the same nest at different depths, as in the case of *N. ephratae* [37]. Interestingly, Jones [13] reported that, in *Nasutitermes costalis*, if the nest is cut open along a plane parallel to the growth direction one observes a tall columnar structure whose description highly resembles our simulation (*b*). As a summary our model appears complex enough to reproduce the variety of structures encountered in real nests as a response to different initial conditions, which suggests that sensitivity to initial conditions also matters in the real growth process.

**Figure 5.**
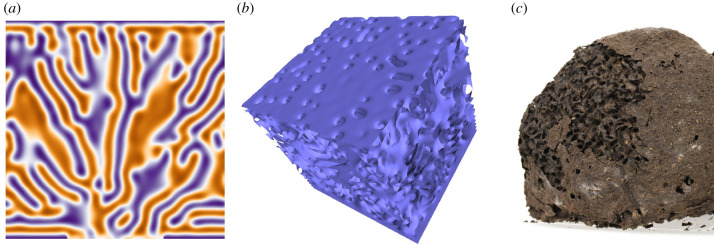
Effect of an *f* = 0 boundary on the nest growth. Panels (*a*) and (*b*) correspond to a two-dimensional vertical cut and a three-dimensional rendering of simulation (*b*) at *t* = 128. The top face of the simulation domain was maintained constant at *f* = 0. One observes that, approaching the forbidden boundary *f* = 0, the simulated nest closes itself, forming a roof which resembles the thin external layer of *Nasutitermes* nests (*c*).

As a second comparison we report that, whenever the growing interface approaches a boundary where *f* is set to 0, the growing tips tend to connect, forming a sort of roof scattered with holes, as is shown in figure 5. Waiting even longer the holes tend to be filled and the connections between the roof and the rest of the structure are progressively eroded. Very interestingly in all the species of arboreal *Nasutitermes*, nests are covered with a uniform thin crust, which in species such as *N. ephratae* happens also to be poorly connected to the bulk structure [14,37].

There is no clear indication in the published literature of what factors control the initiation and termination of a construction activity burst, which is usually extremely rapid compared with the lifetime of a colony [13].

These processes likely involve several external (weather conditions, material availability, etc.) and internal (size and health of the colony) variables that have been completely ignored in our model. However, it is remarkable that our simple model incorporates the boundary sensitivity of the real process. Moreover, we could observe in the field that whenever the external layer of a thriving colony is disrupted, termites rapidly construct a patch that seals the internal structure from the exterior. This suggests that boundary conditions could mimic the maintenance of a non-expanding stage.

At this point, we have already established strong correspondence between our model and real nests: the very convoluted structure (frequent merging and branching), the existence of a typical scale and the sensitivity to external constraints both in space (boundary conditions) and in time (initial conditions). However, all these are mostly qualitative comparisons. Below we define a protocol to make our comparison quantitative. The difficulty of this task is due to the fact that our objects are fundamentally disordered so that a statistical approach is the only possible route. The choice of the relevant quantity to compare may also be discussed, but, coherently with our observations of real nests and with our growth model, the local curvature is the best candidate. The local curvature of a three-dimensional surface in one point *P* can be entirely defined by two principal curvatures *k*_1_ and *k*_2_, which are defined as the minimum and the maximum among the curvatures *k* of all the normal sections, i.e. the planar curves given by the intersection between the surface and a normal plane. Given the osculating circle to such a curve, the value of *k* is the reciprocal of its radius while the sign is positive or negative depending on whether the curve turns in the same or the opposite sense of the local normal vector $\hat{n}$. Equivalently, one can define the mean curvature *H* (which we already encountered in §2) and the Gaussian curvature *Γ*,

5.1 $$H=\frac{k_{1}+k_{2}}{2}\;\Gamma =k_{1}k_{2}.$$

The sign of *Γ* tell us if the surface is more similar to a sphere (*Γ* > 0), a cylinder (*Γ* = 0) or a saddle (*Γ* < 0), while the sign of *H* tells us if we are at the exterior (*H* > 0) or the interior (*H* < 0) of our spherical, cylindrical or saddle-like surface. Finally, *H* = 0 characterizes peculiar saddle-like surfaces which are known as *minimal surfaces* where no interior or exterior can be defined, the two sides being indistinguishable. To define our statistical sample, we take advantage of the discrete representation of our objects. As we defined them, both simulations and real nests are scalar fields defined on a grid of voxels, from where we extract an iso-surface in the form of three-dimensional triangular meshes obtained via the Lewiner Marching Cube [38] algorithm implemented in Python. Our sample space will then be constituted by the set of all surface elements, to which we associate two independent attributes—the mean curvature *H* and the Gaussian curvature *Γ*. These are obtained as a function of the mesh vertex coordinates, as described in electronic supplementary material, section S.II; importantly, these coordinates are first normalized by the typical length given by the FFT in order to make the comparisons consistent. In electronic supplementary material, figure S4, we report a histogram of the frequency of our two observables *H* and *Γ* for a collection of CT scans of real nest fragments, simulations and synthetic data. The distribution of *H* is always peaked and quite symmetric around *H* = 0 while the values of *Γ* are more frequent at *Γ* < 0, which indicates that all our surfaces share the abundance of the saddle-shaped region. Nonetheless, a full characterization of our objects demand that *H* and *Γ* are considered together.

**Figure 6.**
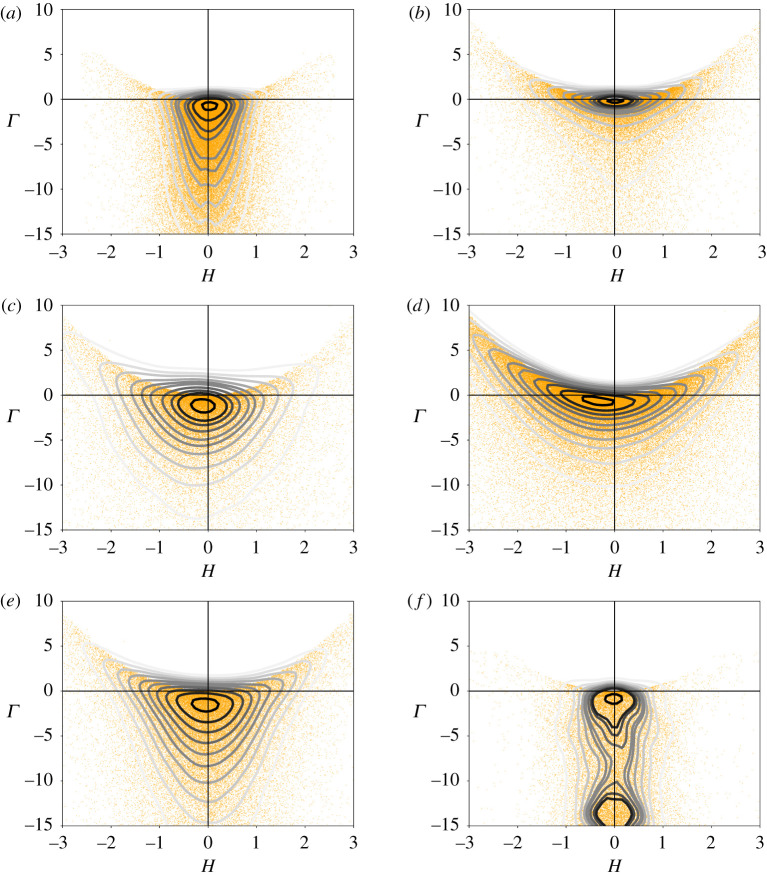
KDE of the frequency of surface mesh elements in the bidimensional space mean curvature (*H*)–Gaussian curvature (*Γ*). Panels (*a*,*b*) and (*c*,*d*) refer to the two simulations and nest fragments, respectively, already analysed in figure 4; (*e*) is a random surface obtained from a volume of white noise which has been band-pass filtered around a certain length and (*f*) is a gyroid which is a minimal surface. Each contour corresponds to the successive 10 percentile of points from a more frequent area (black) to a less frequent area (light grey). Dots represent surface elements (one every five dots is shown).

In figure 6, we have reported the cumulative Gaussian kernel density estimation (KDE; see electronic supplementary material, equation (S2)) of the surface elements in the space (*H*, *Γ*). The regions between two contours indicate where 10% of the total counts occur, from the most frequent area, in black, to the most rare area, in light grey. In the top and central rows, we have reported the two simulations (*a*,*b*) and two fragments of real nests (*c*,*d*) already analysed in figure 4. At the bottom, we consider two synthetic benchmark surfaces which are a random surface (*e*) obtained from a volume of white noise, which has been band-pass filtered around a certain length, and a gyroid (*f*), which is a minimal surface, i.e. *H* = 0 everywhere. One recognizes a strong resemblance in the distribution between simulation (*b*) and nest (*d*). Compared with all the other diagrams one observes that the curvature distribution is pushed towards the centre *H* = *Γ* = 0 and its shape is squeezed in the direction of the *Γ* axis. This distinction is consistent with the one we have made in figure 4 in terms of isotropy/anisotropy, since in simulation (*b*) and nest (*d*) we observe a higher proportion of quasi-flat regions, which is consistent with higher anisotropy. Conversely, simulation (*a*) and nest (*c*) are more isotropic and share a broader distribution along the *Γ* axis, which indicates a higher abundance of saddle-shaped regions and is closer to the curvature distribution of a gyroid (*f*). One also remarks that in nest (*c*) the curvature distribution is more spread along the *H* axis, and it is extremely similar to the curvature distribution of the random surface (*e*). These results suggest that the construction process is intrinsically noisy and that probabilistic rather than deterministic rules control the building behaviour [39,40].

## 6. Discussion

Construction, as performed by humans or other animals, usually involves the interaction between a building agent and some building material under the guidance of innate or learned rules of building. By contrast, growth—as it is known in physics or biology—is an autonomous process where a substrate grows by itself, depending on its interaction with the external environment. Arboreal nests built by *Nasutitermes* termites lie in between these two categories because they originate from the cooperation of thousands of individual workers but the interactions between them are largely guided by the shape of the substrate itself, which makes the nest construction a self-organized process. This justifies our approach of formulating a model where the agents’ behaviour is incorporated in a classical accretion process where the nest growth is focused in the region of high curvature.

By identifying local surface curvature as the only triggering stimulus of termite construction, our model certainly ignores a large part of the complexity of behaviour of termites, and it does not include the possible effects of direct termite interactions, such as traffic and crowding at the building site. Yet, the identification of curvature as an important stigmergic stimulus that can guide termite building activity is well supported in the scientific literature [13,15,16], and our model is sufficient to reproduce some key features of arboreal *Nasutitermes* nests: the intricate structure, the existence of a typical length scale, the sensitivity to boundary and initial conditions and the abundance of saddle-shaped structures of zero mean curvature. On these grounds, we can conclude that, while real termite behaviour could be more complex, a response to curvature alone is in itself sufficient to explain a large part of the complexity observed in arboreal termite nests. The nests of other termite genera such as *Apicotermes* or *Cubitermes* present other structures such as chambers and pillars (reviewed in [10]) that are not naturally produced by our simulations. We can speculate that saddle-like structures such as those observed in arboreal *Nasutitermes* nests could be relatively ancestral features of termite constructions—supported by the fact that such structures are also observed across distant taxa—which can be produced through relatively simple building rules, but other termites have probably evolved more complex building rules that rely on additional stimuli and regulations.

We have designed a minimal nonlinear equation which reproduces a growth process where the growing substrate progressively invades the available space, through a rich dynamics of branching and merging which produces intricate structures similar to *Nasutitermes* nests. We stress here that, while branching is commonly observed in many gradient-driven growth phenomena in both inert [5] and biological matter [4,41], merging is impossible in such simple models [20]. Only recently was merging observed when the gradient was reintroduced on both sides [42]. With regard to our model, we report that merging occurs only in three dimensions, while in two dimensions the symmetry *f* → (1 − *f*) must be broken in order to observe it. We also report that, far from the front of freshly grown substrate, rearrangement of the interface is negligible, although it is permitted. This is consistent with empirical observations [13,43], which report that, in *Nasutitermes*, nest walls are barely rearranged after the initial construction. In a narrow band around the growing front, minor rearrangements are possible; for example, freshly grown material (where *f* > 0.5) can be partially dug later. This is intrinsic to our continuous phase-field approach, but is not in contrast with termites' behaviour, which may keep modifying the wall shape as long as the building material is still humid and easy to mould. In future work, it would be interesting to explore how the pattern produced in the simulations is modified with the addition of an explicit drying effect; for example, coupling our equation with a moisture and temperature field.

All the simulated patterns clearly show the emergence of a characteristic length which corresponds to the one predicted by linear analysis and scales as $1/\sqrt{d}$. This feature constitutes a strong similarity to nest samples collected in the field, which show a characteristic distance between neighbouring walls. In real nests such a distance is comparable to termite body size, which suggests that termites may use their body as a template, as recently proposed for ants [8]. However, our model shows that there is no need to enforce the use of a template to observe the appearance of a typical distance. Interestingly, at linear order our problem is analogous to the buckling instability of a thin elastic plate under a symmetric in-plane compression of magnitude *d*: when *d* is large enough the plate buckles and the compression energy is released in the form of bending energy of the plate. This classical problem has received renewed attention in recent studies that showed how observed bending patterns usually differ from the planar waves predicted by linear analysis [36]. Instead richer patterns appear that are a combination of multiple planar waves oriented in different directions, because of secondary instabilities and nonlinear interactions. A direct comparison between our model and the thin plate problem is not possible because of the different dimensionality and initial conditions. Yet, it is interesting to notice how, similarly to the thin plates problem, in our simulations we never obtain the planar solution but rather observe truly three-dimensional patterns with a variable degree of isotropy depending on the initial conditions. As a general rule, our equation tends to select structures which have a narrow wavelength in one direction and are slightly modulated in the two others.

Incidentally, we observe that nest fragments from different termites species, or even within the same nest, also show a distinct variability in term of isotropy, thus we speculate that initial conditions should be relevant in the growth of real nests as well, but further investigations will be necessary in this regard.

Generated patterns are also very sensitive to boundary conditions and in particular we observe that forbidding the nest expansion (e.g. imposing *f* = 0) at one boundary induces the formation of a uniform layer that isolates the nest from the forbidden boundary. Interestingly, all *Nasutitermes* nests are also covered with an external thin layer that seals the nest from the outside world and is constantly repaired whenever damaged by wetting/drying episodes or other external mechanical stresses, as was observed both in the field and in the laboratory. A forbidden boundary does not necessarily correspond to the existence of a solid boundary but could correspond, for instance, to a threshold level of pheromone emanating from the colony, as in the modelling study by Ocko *et al.* [44] or a steep gradient in air humidity corresponding to the transition between the inner humid ambient of the nest and the dry exterior, as recently suggested by the experimental study of Soar *et al.* [45]. In any case, we find it relevant that our simple construction model responds in a consistent way in terms of expressed morphology. Future empirical studies would be needed to identify the releaser stimuli that trigger the initiation and termination of nest expansion.

Both the simulated and scanned surfaces show large portions of zero mean curvature (*H*) and non-positive Gaussian curvature (*Γ*), which corroborates the hypothesis that curvature is the main driving mechanism in construction. In fact, if growth happens in the region of high mean curvature, we expect that a stationary or quasi-stationary configuration should correspond to one where local mean curvature is low or null. Consistently, whenever the structure is saddle-shaped, highly connected and isotropic, we find that Gaussian curvature is negative, *Γ* < 0. Alternatively, we observe in both simulations and real nests the existence of sheet-like structures of the type (*H* ∼ 0, Γ≲0), which corresponds to the emergence of a clear anisotropy. We also remark that curvature distribution of isotropic nests resembles that of a random synthetic surface with a typical length scale, which confirms the absence of a global predetermined architecture. This is consistent with a construction process driven primarily by local information, as we implemented in our model.

While nest structure is primarily random on the local scale, we already observed that nest fragments can have different degrees of internal anisotropy and distinctive features such as the external envelope. Owing to the relatively small size of our samples, we do not have a detailed characterization of nest topology and connectivity on the global scale and the question remains open: are the observed topology and connectivity emerging properties of the same local construction mechanism—as in the present model—or must they be enforced through additional global rules? In addition, we plan to more thoroughly investigate the stimuli that trigger the beginning or the end of a construction stage. Previous literature is all but conclusive in this regard but we expect that activation and inhibition should be related to the colony size, which can be possibly translated to the abundance of some auxiliary field, such as relative humidity, CO_2_ or a pheromone. To this aim, we will couple our model with some auxiliary field that is constrained to diffuse or convect in the nest galleries and explore whether we can observe significant time modulation in the nest expansion.

In the general context of accretion processes, we believe that our equation may be adapted to other phenomena where a growth rate sensitive to curvature and a local smoothing process coexist, which is encouraged by the simplicity of the equation and the richness of the expressed patterns. While our model was primarily formulated to describe the morphogenesis of arboreal termite nests, saddle-shaped structures with a characteristic scale are also observed in other biological systems, ranging from the nests of other insects not directly related to termites (e.g. *Lasius fuliginosus*) to the micro-structure of butterfly wings [46]. It is possible that our model could also provide insight into the formation of these other structures. The scientific literature on social insects has often focused on agent-based models as a tool to describe the behaviour of individual insects and the emerging organization of the colony. We are confident that simple ‘stigmergic’ models like the one developed here have a great potential to explain not only how structures are built, but also how transitions between different nest plans can be triggered by small changes in the building parameters or environmental conditions. In relation to real nests, such changes could correspond to transitions between nest plans that emerge across phylogenies and could also reflect adaptive changes in response to different habitats.

## Supplementary Material

Supplementary material

## Data accessibility

Finite difference code to simulate the growth equation is available at the following link: https://github.com/oiluigio/JRSI_2020_termite_nest Initial condition fields are available from Figshare: https://figshare.com/articles/dataset/A_growtḣmodel_driven_by_curvature_reproduces_geometric_features_of_arboreal_termite_nests/12570680
