# Supplementary material for: A growth model driven by curvature reproduces geometric features of arboreal termite nests
Source: J R Soc Interface. 2020 Jul 22;17(168):20200093. doi: 10.1098/rsif.2020.0093 (PMC7423439; doi:10.1098/rsif.2020.0093)
Supplement: Supplementary material [file rsif20200093supp1.pdf]

Supplementary material: a growth model driven by curvature reproduces geometric features of arboreal termite nests

G. Facchini[1], A. Lazarescu[2], A. Perna[1], and S. Douady[3]

[1]Life Sciences Department, University of Roehampton, London, UK
[2]Institut de Recherche en Mathématique et Physique, UCLouvain, Louvain-la-Neuve, Belgium
[3]Laboratoire Matière et Systèmes Complexe, Université Paris Diderot, Paris, France

S.I Linear and scale analysis

In its simplified form the growth equation (3) can be easily linearised around $f_0 = 0.5$ and solved in Fourier space with solutions of the form $f = f_0 + \hat{f} \exp[i\boldsymbol{k} \cdot \boldsymbol{x} + \sigma t]$ which gives the following dispersion relation:

$$\sigma(k) = \tilde{f} d k^2 - k^4 \tag{S1}$$

where for simplicity we wrote $|\boldsymbol{k}| = k$ and $\tilde{f} = f_0(1 - f_0)$. The dispersion relation is shown in figure S1. As d is strictly positive, $\sigma(k)$ changes sign in $k_0 = (\tilde{f}d)^{1/2}$ and has a local maximum at $k_{max} = (\tilde{f}d/2)^{1/2}$: equation (3) is linearly unstable with the most unstable length at $\lambda_{max} = 2\pi/k_{max}$, while all the small scale disturbances beyond the cutoff length $\lambda_0 = 2\pi/k_0$ are damped. Note that $k_0 > 0$ independently of d, thus the system is formally always unstable. Nonetheless a threshold is fixed by the smallest k which can appear in a given domain of size L which is $k = 2\pi/L$. The instability threshold d_c is then fixed by the size L of the domain, with $d_c = (2\pi/L)^2/\tilde{f}$ the marginal condition. In figure S2 we report the fast Fourier transform performed on the scalar field associated to two simulations and the computer-tomography images of two nests collected from the field. One observes the presence of a local maximum which denotes the emergence of a characteristic length scale.

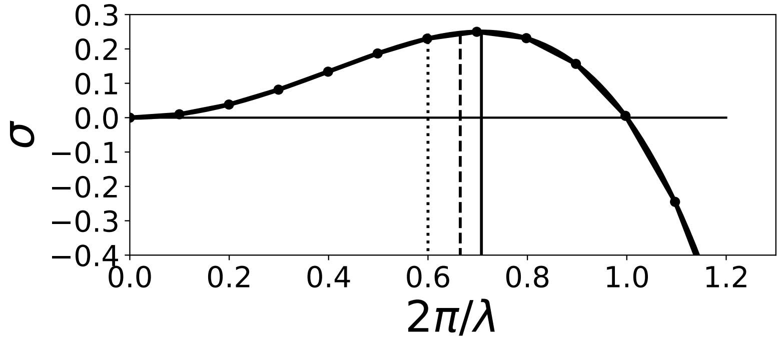

Figure S1: Dispersion relation S1 for small-amplitude perturbations, here $\tilde{f} = 0.25$ and $d = 4$. The solid line indicates the most unstable length scale and the corresponding growth rate $\sigma = \sigma_{max}$ is used to rescale time in our simulations. Dashed and dotted lines indicate the typical length that appears in simulations (a) and (b) reported in figure 4. Black dots refer to lengths that can fit in our simulation box.

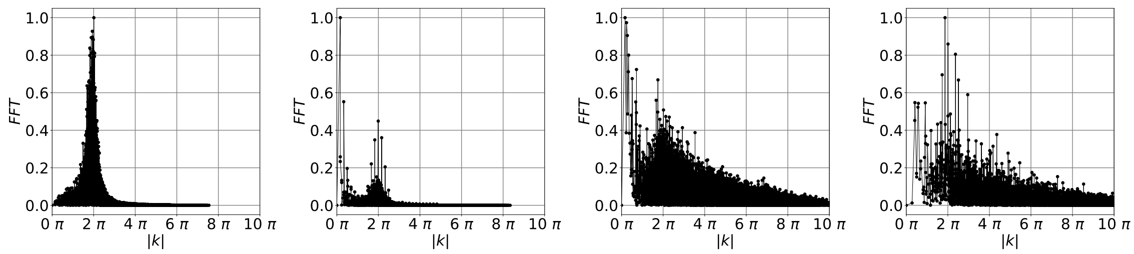

Figure S2: Amplitude spectrum of the fast fourier transform (FFT) of the scalar field associated to simulations (a,b) and CT-scans of real nests (c,d) shown at the top of figure 4. The local maxima correspond to the emergent characteristic length scales which are reported in figure 4. Wave numbers k are normalized by the characteristic length scale. Note that FFT of real nests are performed on the raw version of CT-scans.

S.II Segmentation and curvature statistical distribution

In this section we explain how we obtain surfaces from a scalar field and construct the curvature distributions shown in figure S4 and 6.

The protocol includes five steps: (i) smoothing and segmentation of the original scalar field, (ii) isosurface extraction, (iii) smoothing of the surface and (iv) computation of curvature, (v) statistical analysis of curvature distribution.

The first step only concerns nest samples collected in the field, in fact CT images must be pre-processed to remove some of the small scale roughness of the nests and the noise introduced by the micro-CT scanner. The pre-process include 4 stages (a,b,c,d) which are summarised in figure S3 where we compare four sample slices at the beginning and at the end of the pre-process. First (a), original CT images are cropped to the area of interest and their contrast/luminosity enhanced using the software *imageJ*. Then (b), we filter the original greyscale images by eliminating 10 to 20% of the highest Fourier components, which conveniently reduces the size of our data and removes isolated noise voxels due to acquisition noise or small spurious particles inside the nests (dust, dead termites, etc.). We follow (c) this operation by applying a Gaussian filter of width $\sigma = 1$, mainly to remove the ripples introduced by the previous sharp low-pass filtering operation. After this initial blurring operation, we (d) binarise our stack of images and we apply a second Gaussian filter of width $\sigma = 1$. This final smoothing operation allows having a smooth transition between walls and galleries while having a uniform value $f = 1$ deep in the walls and $f = 0$ in the middle of a gallery. Note that the threshold for segmentation must be chosen by hand and adjusted in order to preserve the topology of the original scanner which is inspected comparing slices of the original stack to the processed ones.

The second step is the isosurface extraction, that results in the production of a three-dimensional triangular mesh which represents the iso-contour surface of the scalar field at a given threshold f_0. We use the efficient Lewiner implementation of the Marching Cubes algorithm [*S1*] provided by Python scikit-image library [*S4*]. This essentially performs an iteration over all the voxels of the considered volume and draws a new surface element within all the voxels where there is at least one vertex with $f > f_0$ and one with $f < f_0$.

As a third step we compute the mean curvature H and the Gaussian curvature Γ at each vertex of the mesh using the algorithm proposed by Meyer et al [*S2*]. Mean curvature basically corresponds to the sum of all edges connecting the considered vertex to its neighbors, while Gaussian curvature is essentially the sum of the angles

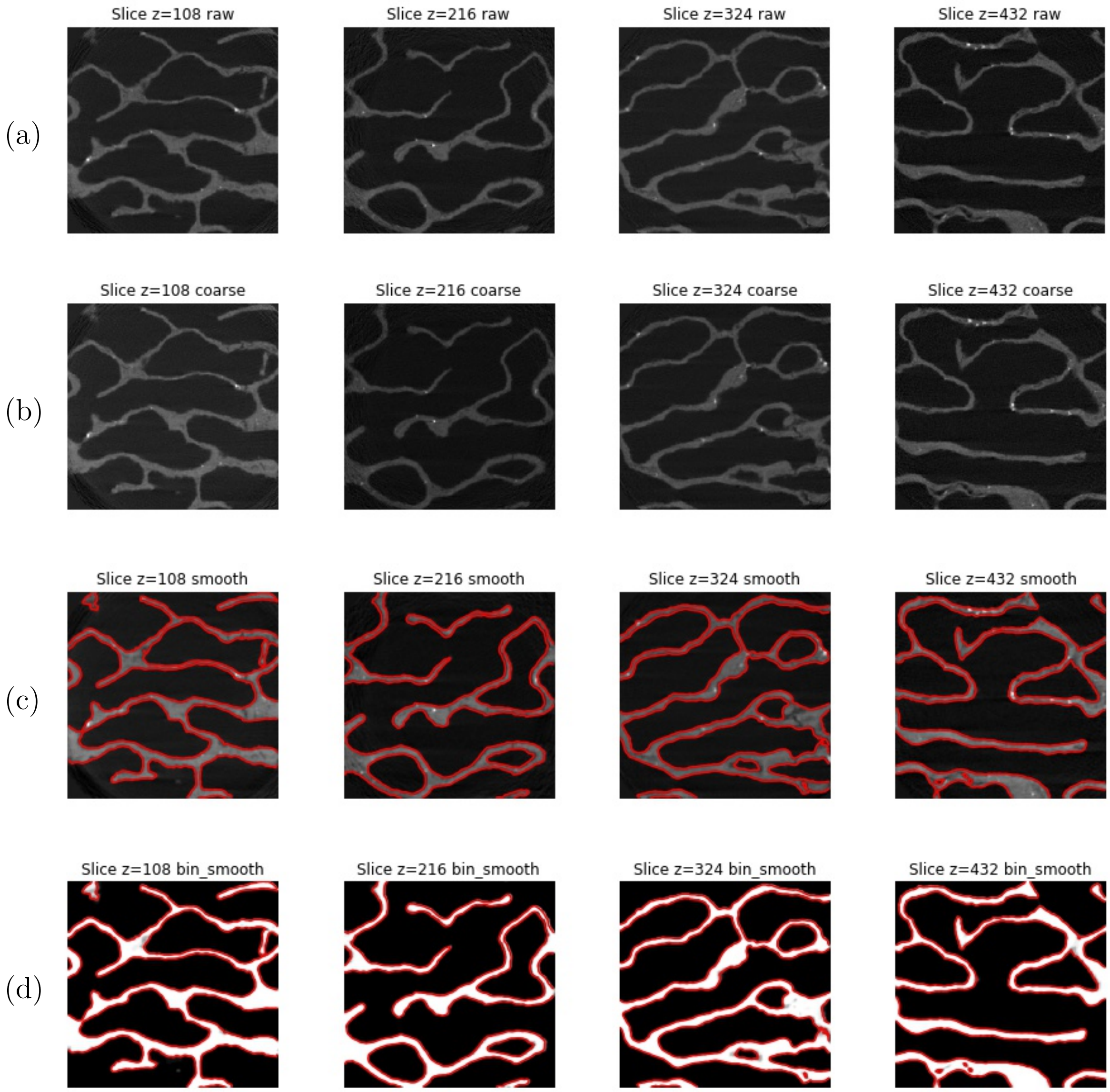

Figure S3: Illustration of the different steps adopted to reduce noise on CT-scans. (a) at this stage images have been slightly cropped and the contrast/luminosity have been enhanced using *imageJ*. (b) Fourier coarsening, the 20% highest Fourier modes have been removed. (c) 1st Gaussian filter, images are smoothed with a Gaussian filter of width $\sigma = 1$, and a first threshold is chosen (red line). (d) 2nd Gaussian filter, images are binarized using the first threshold and smoothed with a second Gaussian filter of width $\sigma = 1$. A second threshold is chosen which serves to extract the isosurface (red line).

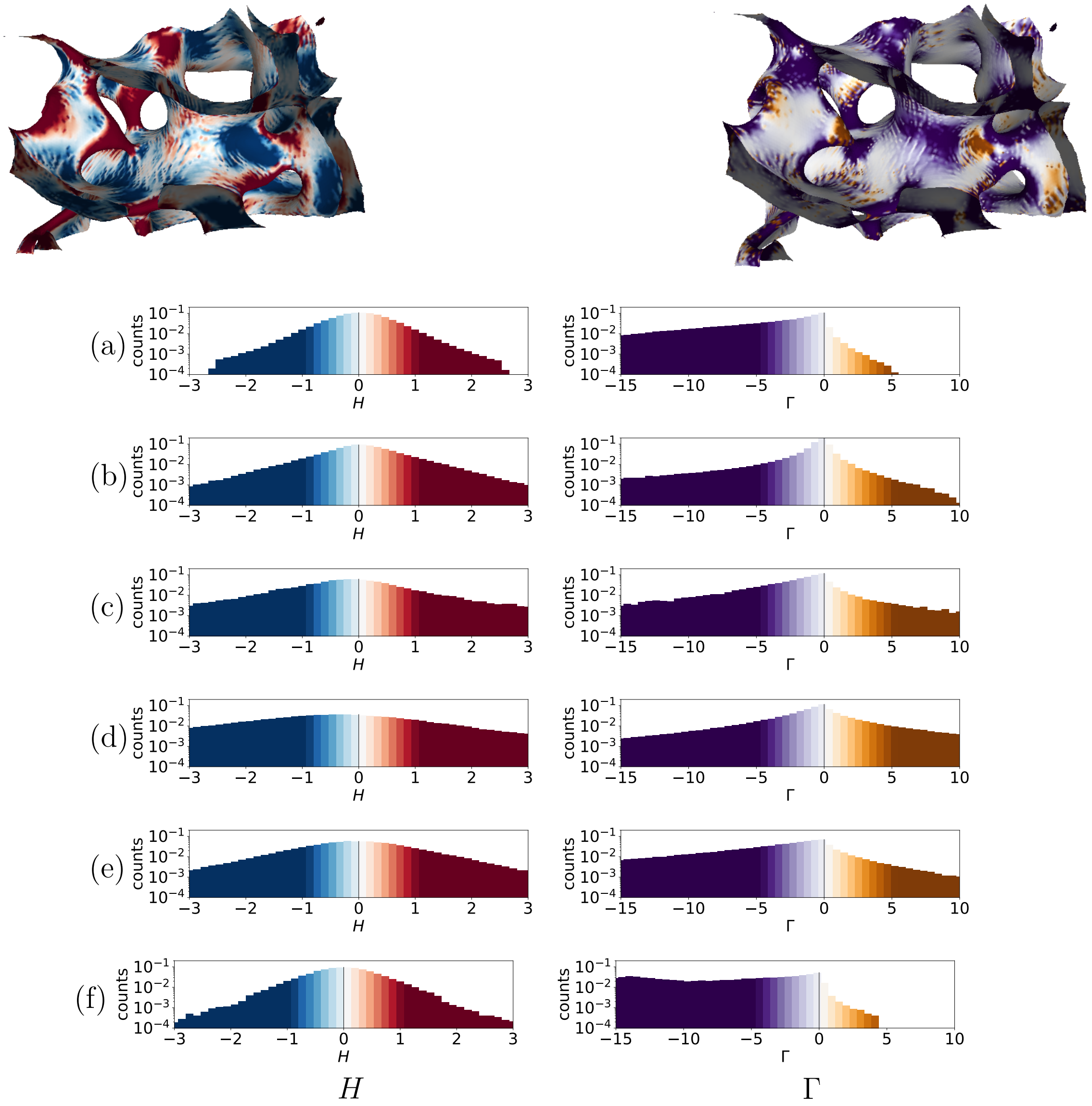

Figure S4: Normalised frequency of mean curvature H (left) and Gaussian curvature Γ (right) for different surfaces, (a): simulation initiated with white noise, (b): simulation initiated with a nest-like seed, (c): nest fragment from Guyane-*N.ephratae*, (d): nest fragment from New South Wales (AUS)-*N. walkeri*, (e): pass-band filtered noise, (f): gyroid minimal surface. The 3D renderings at the top represent the surface of nest fragment (c) coloured according to the local value of H (left) and Γ (right).

between these edges and those connecting the neighbor vertices, weighted by the area of the corresponding face. Prior to this computation the coordinates of all vertices are normalised by the typical length given by the FFT of the original scalar field: with this choice a surface element whose curvature is $(H, \Gamma) = (1, 1)$ corresponds to an element of spherical surface of radius equal to the typical distance between the center of two walls/tunnels. In addition, as we are using a first-neighbors algorithm, we expect the curvature computation to be extremely sensitive to the mesh roughness which results from the discrete nature of the original scalar field (sampled in discrete voxels). In order to remove this small scale noise (which is not relevant to the actual characteristic scale of the structure), each triangular mesh was smoothed averaging each vertex coordinates with the coordinates of neighboring vertices with all vertices taken with the same weight. Our averaging algorithm is unconstrained which means that smoothed meshes tend to shrink and have a smaller volume than the original one. Nevertheless we observed that even after 20 iterations the shrinking effect was negligible and averaged around 2%. Note that the averaging step is critical, since on one hand we want to get rid of small scale/high curvature disturbances, and on the other side we ultimately want surface curvature to characterize our objects, for example we must avoid modifying the surface too much. To this aim we verified that by repeating the smoothing operation several times, curvature properties tend to converge as it is shown in figure S5 and S6. Finally it is worth to notice that curvature is measured locally, thus it is important that scans are well resolved. This condition was satisfied by our scans and was verified comparing the curvature distributions of the original surfaces with their coarse-grained version, where half of the Fourier modes were suppressed. The results are shown in figure S7, we observe that for the two nest fragments presented in this work, reducing the resolution by a half poorly affects the curvature distribution, which means that to the purpose of curvature analysis our scans are well resolved.

The last steps consist in producing the curvature distribution of the analysed surfaces. In figure S4 we report the normalised frequency of occurrence for H and Γ in the form of histograms for a sample of two different simulations (a,b), two different nest fragments (c,d), and two benchmark synthetic surfaces (e,f) where (e) is a random surface obtained by segmentation of a volume of band-pass filtered white noise and (f) is a minimal surface (i.e. $H = 0$ everywhere) called gyroid. Subsequently, we present the same data in the two dimensional diagram (H, Γ) and estimate the probability density ρ_{kde} with a Gaussian kernel which is defined as below:

$$\rho_{kde}(\boldsymbol{x}) = \frac{1}{nh} \sum_{i=1}^{n} e^{\frac{|\boldsymbol{x}-\boldsymbol{x}_i|}{2h^2}}, \quad \text{(S2)}$$

where $\boldsymbol{x}$ is one generic point in the plane (H, Γ) while $\boldsymbol{x}_{i=1,n}$ are all the surface elements. This tool is provided by the Python scipy library and the bandwidth h is estimated with the rule-of-thumb [*S3*]. The cumulative probability density associated with ρ_{kde} is shown in figure 6, S5, S6 and S7 where each contour corresponds to the successive ten percentiles from the most frequent area (black) to the least frequent area (grey). Dots correspond to the raw data before the kernel density estimation is realised. One observes that raw data are constrained to remain below the parabola $\Gamma = H^2$ which is consistent with the definition of H and Γ given in equation (5). Note that kernel density estimation smoothens this boundary which explains why contour lines (but no dots) extend to the forbidden region.

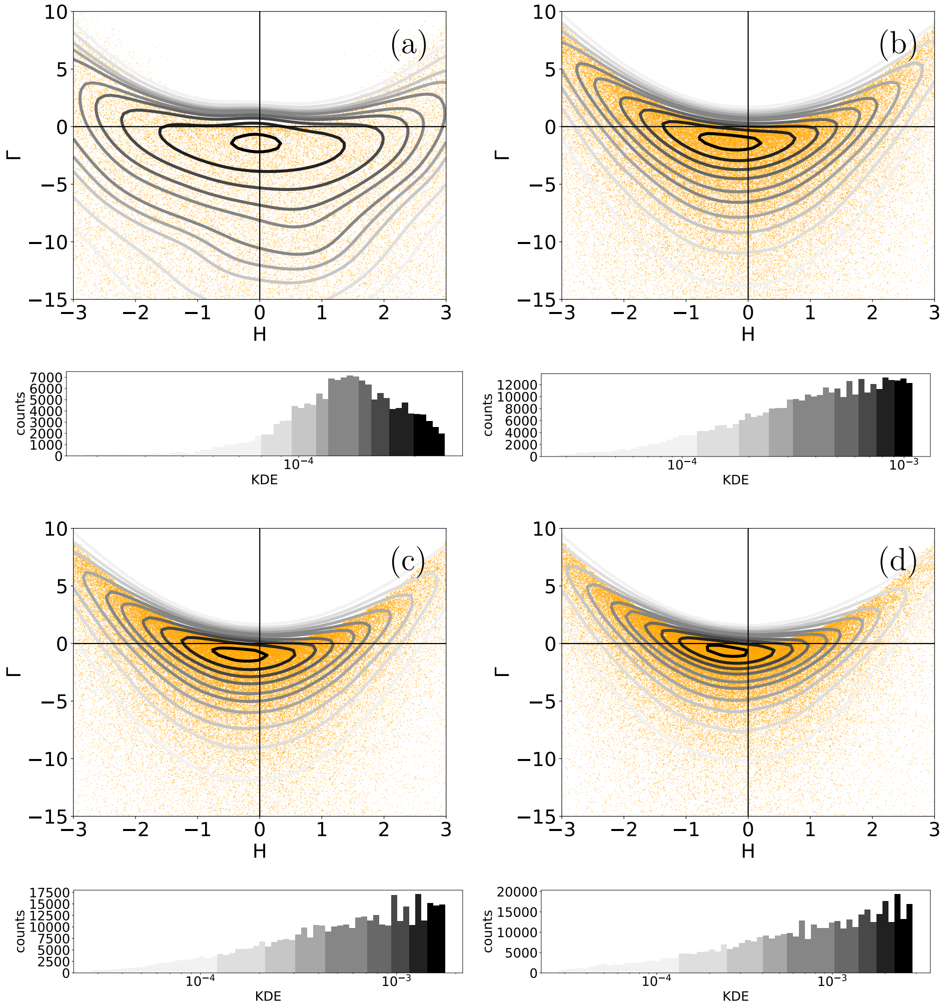

Figure S5: Curvature distribution for a nest fragment of *Nasutitermes walkeri*, where Laplacian smoothing algorithm has been applied 0 (a), 5 (b), 10 (c), and 20 (d) times. The distribution remains relatively stable after 10 iterations.

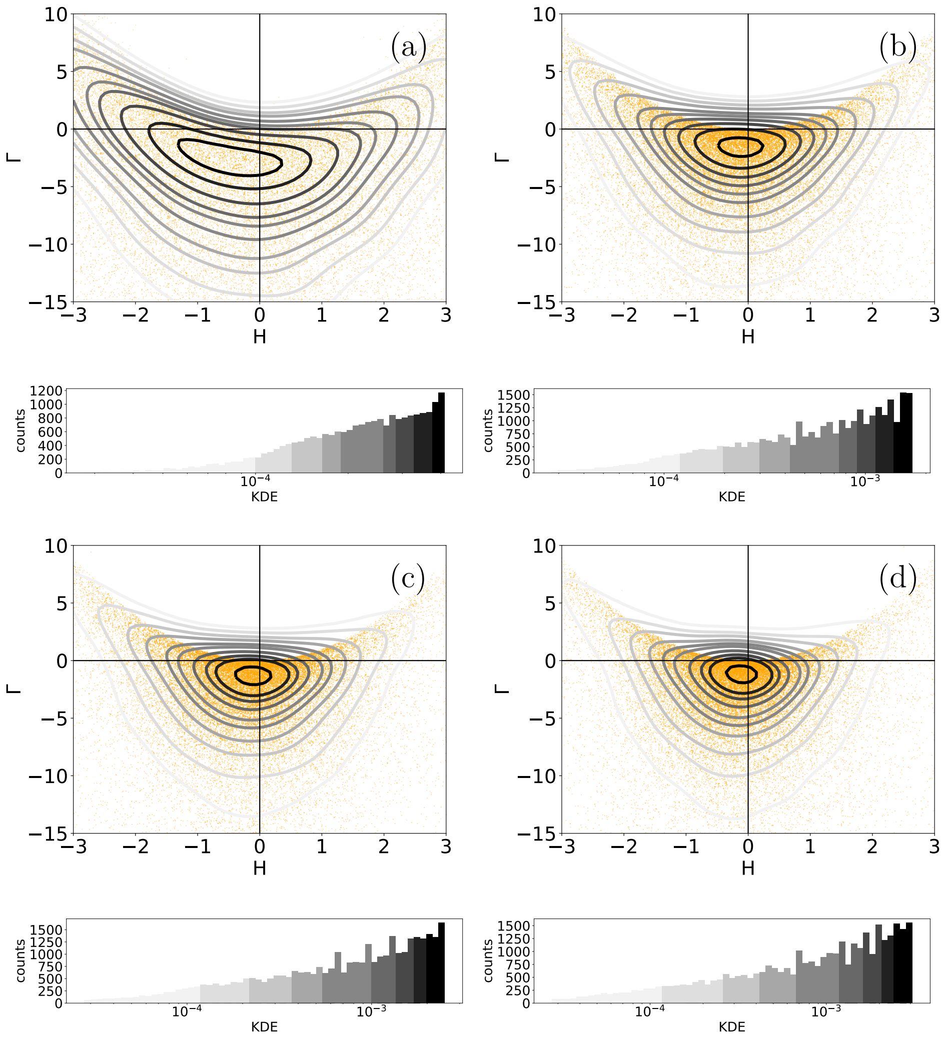

Figure S6: Curvature distribution for a nest fragment of *Nasutitermes ephratae*, where Laplacian smoothing algorithm has been applied 0 (a), 5 (b), 10 (c), and 20 (d) times. The distribution remains relatively stable after 10 iterations.

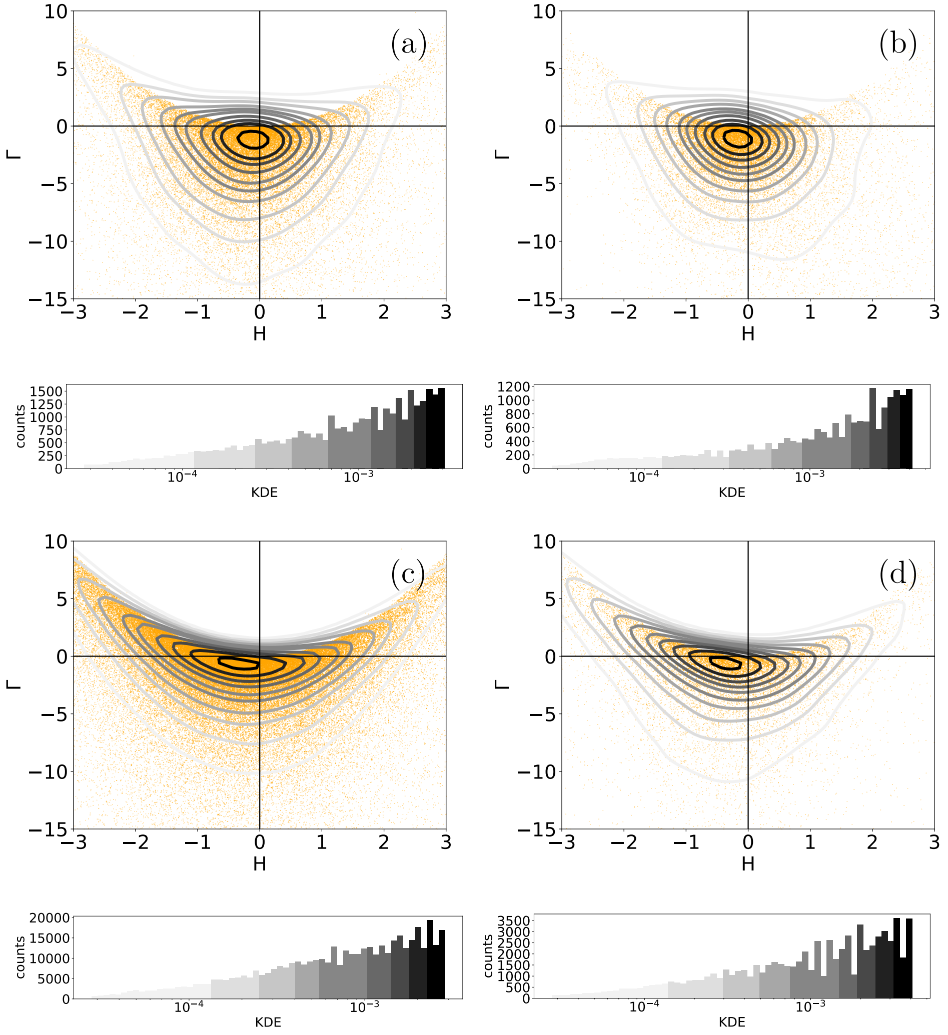

Figure S7: Top: curvature distributions for a nest fragment of *Nasutitermes ephratae*, where 10% (a) and 50% (b) of Fourier modes were suppressed. Bottom: curvature distributions for a nest fragment of *Nasutitermes walkeri*, where 20% (c) and 50% (d) of Fourier modes were suppressed. Note that the diagrams on the left are the same presented in the paper (panel (c) and (d) respectively in Fig. 4 and Fig. 6, and those on the right correspond to half the resolutions of the original scans.

References

[S1] T. Lewiner, H. Lopes, A. W. Vieira, and G. Tavares. Efficient implementation of marching cubes' cases with topological guarantees. *Journal of Graphics Tools*, 8(2):1–15, 2003. doi: 10.1080/10867651.2003.10487582.

[S2] M. Meyer, M. Desbrun, P. Schröder, and A. H. Barr. Discrete Differential-Geometry Operators for Triangulated 2-Manifolds. In G. Farin, H.-C. Hege, D. Hoffman, C. R. Johnson, K. Polthier, H.-C. Hege, and K. Polthier, editors, *Visualization and Mathematics III*, pages 35–57. Springer Berlin Heidelberg, Berlin, Heidelberg, 2003.

[S3] D. Scott. *Multivariate Density Estimation: Theory, Practice, and Visualization.* A Wiley-interscience publication. Wiley, 1992.

[S4] S. van der Walt, J. L. Schönberger, J. Nunez-Iglesias, F. Boulogne, J. D. Warner, N. Yager, E. Gouillart, T. Yu, and the scikit-image contributors. scikit-image: image processing in Python. *PeerJ*, 2:e453, 6 2014. doi: 10.7717/peerj.453.
